# Diisopropyl­ammonium hydrogen phenyl­phospho­nate

**DOI:** 10.1107/S1600536812041086

**Published:** 2012-10-06

**Authors:** Modou Sarr, Mouhamadou Sembene Boye, Aminata Diasse-Sarr, Arnaud Grosjean, Philippe Guionneau

**Affiliations:** a Laboratoire de Chimie Minerale et Analytique, Département de Chimie, Faculté des Sciences et Techniques, Université Cheikh Anta Diop, Dakar, Senegal; b CNRS, Universite de Bordeaux, ICMCB, 87 Avenue du Dr A. Schweitzer, Pessac F-33608, France

## Abstract

In the title salt, [(CH_3_)_2_CH]_2_NH_2_]^+^·[C_6_H_5_PO_2_(OH)]^−^, the anions are linked by pairs of O—H⋯O hydrogen bonds, forming inversion dimers. These dimers are bridged by the cations *via* N—H⋯O hydrogen bonds, leading to a three-dimensional structure.

## Related literature

For crystal structures of closely related compounds, see: Diop *et al.* (2012 ▶); Beckmann *et al.* (2003 ▶).
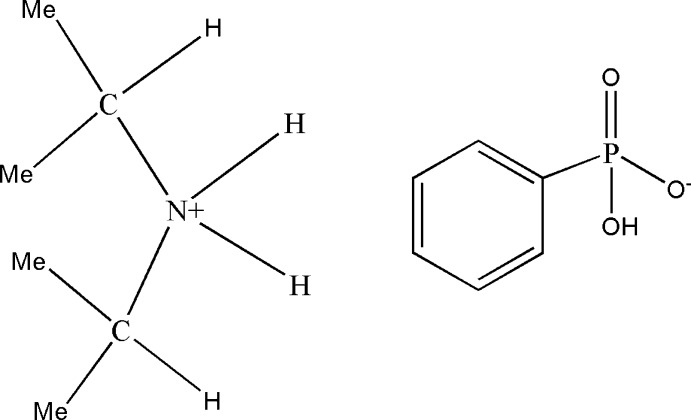


## Experimental

### 

#### Crystal data

C_6_H_16_N^+^·C_6_H_6_O_3_P^−^
*M* *_r_* = 259.28
Monoclinic, 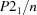

*a* = 11.9166 (2) Å
*b* = 9.0982 (1) Å
*c* = 12.8539 (1) Å
β = 101.013 (1)°
*V* = 1367.95 (3) Å^3^
*Z* = 4
Mo *K*α radiation
μ = 0.20 mm^−1^
*T* = 293 K
0.88 × 0.63 × 0.25 mm

#### Data collection

Nonius KappaCCD diffractometer
Absorption correction: multi-scan (*SCALEPACK*; Otwinowski & Minor, 1997 ▶) *T*_min_ = 0.845, *T*_max_ = 0.952
7738 measured reflections
3990 independent reflections
3626 reflections with *I* > 2σ(*I*)
*R*_int_ = 0.013

#### Refinement

*R*[*F* ^2^ > 2σ(*F* ^2^)] = 0.029
*wR*(*F* ^2^) = 0.080
*S* = 1.03
3990 reflections
243 parameters
All H atoms refined
Δρ_max_ = 0.38 e Å^−3^
Δρ_min_ = −0.33 e Å^−3^

### 

Data collection: *COLLECT* (Hooft, 1998 ▶); cell refinement: *HKL* *DENZO* (Otwinowski & Minor, 1997 ▶); data reduction: *SCALEPACK* (Otwinowski & Minor, 1997 ▶); program(s) used to solve structure: *SHELXS97* (Sheldrick, 2008 ▶); program(s) used to refine structure: *SHELXL97* (Sheldrick, 2008 ▶); molecular graphics: *ORTEP-3* (Farrugia, 1997 ▶); software used to prepare material for publication: *publCIF* (Westrip, 2010 ▶).

## Supplementary Material

Crystal structure: contains datablock(s) I, global. DOI: 10.1107/S1600536812041086/pv2589sup1.cif

Structure factors: contains datablock(s) I. DOI: 10.1107/S1600536812041086/pv2589Isup2.hkl

Additional supplementary materials:  crystallographic information; 3D view; checkCIF report

## Figures and Tables

**Table 1:** Hydrogen-bond geometry (Å, °)

*D*—H⋯*A*	*D*—H	H⋯*A*	*D*⋯*A*	*D*—H⋯*A*
N1—H17⋯O3	0.900 (14)	1.960 (14)	2.8510 (10)	170.3 (12)
O2—H6⋯O3^i^	0.847 (19)	1.744 (19)	2.5895 (10)	177.4 (19)
N1—H16⋯O1^ii^	0.916 (15)	1.764 (15)	2.6782 (10)	176.7 (13)

Symmetry codes: (i) 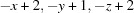; (ii) 
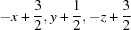
.

**Fig. 1.**
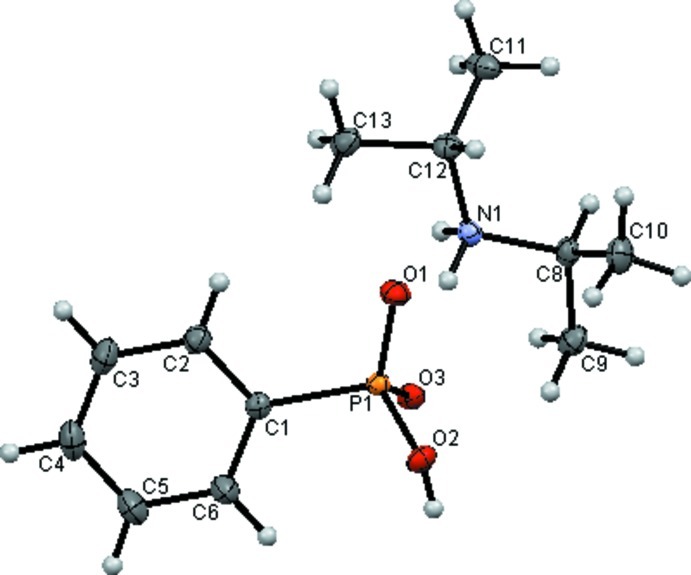
The assymmetric unit of the title salt. Displacement ellipsoids are drawn at the 50% probability level.

**Fig. 2.**
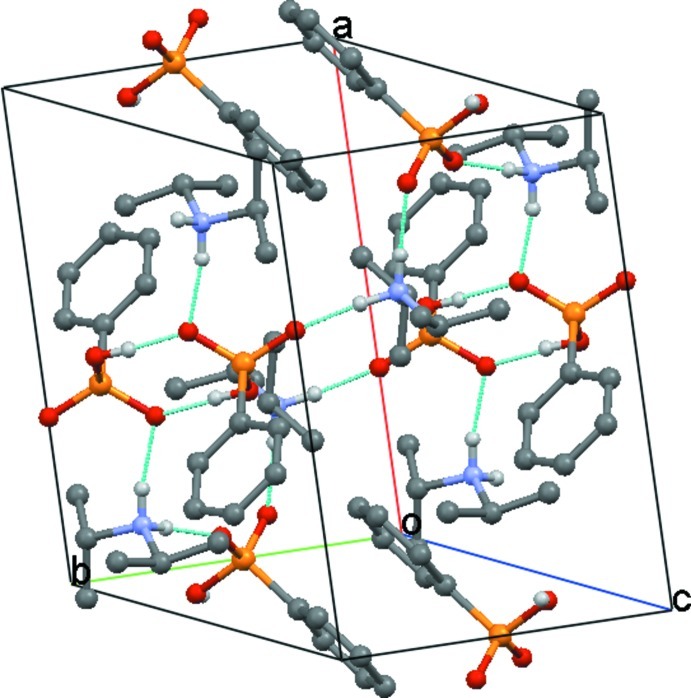
A view of the O—-H···O and N—H···O hydrogen bonds (light-blue colored lines) in the crystal structure of the title compound. H atoms non-participating in hydrogen-bonding were omitted for clarity.

## supplementary crystallographic information

### Comment

In the assymmetric unit of the title salt (Fig. 1), the anion, hydrogen phenylphosphonate (PhPO_3_H^-^), adopts a tetrahedral geometry, with three oxygen atoms and a benzene group with O—P—O and O—P—C angles in the ranges 109.58 (4) - 115.20 (4) and 105.24 (4) - 108.51 (4)°, respectively. Two P—O distances in the anion are close, (P1—O1 = 1.4932 (7) and P1—O3 = 1.5191 (7) Å) indicating the presence of extensive π-delocalization of the P═O double bonds. The bond distance P1—O2 (1.5809 (7) Å for P—OH bond) is significantly longer than the other two P—O bonds. The P—O bond distances in the title salt agree very well with the corresponding bond distances reported in closely related compounds (Diop *et al.*, 2012; Beckmann *et al.*, 2003).

In the crystal, the anions are connected by O2—H6···O3 hydrogen bonds forming dimers about inversion centers. These pairs are then bridged through cations *via* N—H···O hydrogen bonds leading to a three-dimensional structure (Tab. 1 & Fig. 2).

### Experimental

The title compound was synthesized by mixing [(CH_3_)_2_CH]_2_NH and PhPO_3_H_2_ in water (1/1 ratio). The precipitate obtained was filtered. The crystals suitable for X-ray crystallographic analysis were grown from a solution of water by slow evaporation at room temperature.

### Refinement

All H atoms were located from difference maps and were allowed to refine freely with *U*_iso_.

### Crystal data

**Table:**

C_6_H_16_N^+^·C_6_H_6_O_3_P^−^	*F*(000) = 560
*M**_r_* = 259.28	*D*_x_ = 1.259 Mg m^−^^3^
Monoclinic, *P*2_1_/*n*	Mo *K*α radiation, λ = 0.71073 Å
Hall symbol: -P 2yn	Cell parameters from 4223 reflections
*a* = 11.9166 (2) Å	θ = 0.4–30.0°
*b* = 9.0982 (1) Å	µ = 0.20 mm^−^^1^
*c* = 12.8539 (1) Å	*T* = 293 K
β = 101.013 (1)°	Prism, colourless
*V* = 1367.95 (3) Å^3^	0.88 × 0.63 × 0.25 mm
*Z* = 4	

### Data collection

**Table:**

Nonius KappaCCD diffractometer	3990 independent reflections
Radiation source: fine-focus sealed tube	3626 reflections with *I* > 2σ(*I*)
Graphite monochromator	*R*_int_ = 0.013
φ scans, and ω scans with κ	θ_max_ = 30.1°, θ_min_ = 3.2°
Absorption correction: multi-scan (*SCALEPACK*; Otwinowski & Minor, 1997)	*h* = −16→16
*T*_min_ = 0.845, *T*_max_ = 0.952	*k* = −12→12
7738 measured reflections	*l* = −18→18

### Refinement

**Table:**

Refinement on *F*^2^	Secondary atom site location: difference Fourier map
Least-squares matrix: full	Hydrogen site location: inferred from neighbouring sites
*R*[*F*^2^ > 2σ(*F*^2^)] = 0.029	All H-atom parameters refined
*wR*(*F*^2^) = 0.080	*w* = 1/[σ^2^(*F*_o_^2^) + (0.0397*P*)^2^ + 0.4747*P*] where *P* = (*F*_o_^2^ + 2*F*_c_^2^)/3
*S* = 1.03	(Δ/σ)_max_ = 0.001
3990 reflections	Δρ_max_ = 0.38 e Å^−^^3^
243 parameters	Δρ_min_ = −0.33 e Å^−^^3^
0 restraints	Extinction correction: *SHELXL*, Fc^*^=kFc[1+0.001xFc^2^λ^3^/sin(2θ)]^-1/4^
Primary atom site location: structure-invariant direct methods	Extinction coefficient: 0.013 (2)

### Special details

**Table:**

Experimental. The analytical data - % calculated (% found): C: 55.59 (55.64); H: 8.55 (8.91); N: 5.40 (5.58).Infrared data (cm^-1^) [br= broad; s= strong; m=medium]2694br νNH; 1148*m*, 1129 s, 1059, 1027*m*νPO_3_; 898 s δPO_3_; 752 m νPC; 708 s, 696 s νPh.Melting point = 437–438 K
Geometry. All s.u.'s (except the s.u. in the dihedral angle between two l.s. planes) are estimated using the full covariance matrix. The cell s.u.'s are taken into account individually in the estimation of s.u.'s in distances, angles and torsion angles; correlations between s.u.'s in cell parameters are only used when they are defined by crystal symmetry. An approximate (isotropic) treatment of cell s.u.'s is used for estimating s.u.'s involving l.s. planes.
Refinement. Refinement of *F*^2^ against ALL reflections. The weighted *R*-factor *wR* and goodness of fit *S* are based on *F*^2^, conventional *R*-factors *R* are based on *F*, with *F* set to zero for negative *F*^2^. The threshold expression of *F*^2^ > 2σ(*F*^2^) is used only for calculating *R*-factors(gt) *etc*. and is not relevant to the choice of reflections for refinement. *R*-factors based on *F*^2^ are statistically about twice as large as those based on *F*, and *R*- factors based on ALL data will be even larger.

### Fractional atomic coordinates and isotropic or equivalent isotropic displacement parameters (Å^2^)

**Table:**

	*x*	*y*	*z*	*U*_iso_*/*U*_eq_	
P1	0.966159 (19)	0.40247 (2)	0.846319 (17)	0.01271 (8)	
O2	1.04330 (6)	0.32302 (7)	0.94386 (5)	0.01729 (14)	
N1	0.66048 (7)	0.51553 (9)	0.78554 (6)	0.01430 (15)	
C1	1.06532 (7)	0.49048 (10)	0.77593 (7)	0.01453 (17)	
C6	1.14485 (9)	0.59178 (11)	0.82778 (8)	0.0222 (2)	
C12	0.62928 (8)	0.41946 (10)	0.68876 (8)	0.01742 (18)	
C13	0.69808 (9)	0.47210 (12)	0.60787 (8)	0.0225 (2)	
C2	1.06337 (9)	0.46114 (11)	0.66912 (8)	0.01979 (19)	
C4	1.21864 (9)	0.63037 (13)	0.66783 (9)	0.0270 (2)	
C3	1.13968 (9)	0.53095 (13)	0.61530 (9)	0.0256 (2)	
C5	1.22118 (10)	0.66111 (13)	0.77426 (10)	0.0287 (2)	
O1	0.89926 (6)	0.29081 (8)	0.77497 (5)	0.02025 (15)	
C8	0.60257 (8)	0.48352 (11)	0.87717 (8)	0.01757 (18)	
C9	0.64492 (9)	0.59615 (12)	0.96314 (8)	0.0215 (2)	
C11	0.50164 (9)	0.42810 (13)	0.64537 (9)	0.0245 (2)	
C10	0.62574 (10)	0.32619 (12)	0.91508 (9)	0.0257 (2)	
O3	0.89542 (5)	0.52144 (7)	0.88605 (5)	0.01657 (14)	
H6	1.0624 (16)	0.376 (2)	0.9984 (15)	0.054 (5)*	
H17	0.7362 (12)	0.5109 (14)	0.8110 (11)	0.022 (3)*	
H16	0.6429 (12)	0.6104 (16)	0.7649 (11)	0.028 (3)*	
H21	0.5218 (11)	0.4980 (14)	0.8513 (10)	0.018 (3)*	
H18	0.6510 (11)	0.3197 (15)	0.7094 (10)	0.020 (3)*	
H10	0.6767 (12)	0.5719 (16)	0.5864 (11)	0.029 (4)*	
H7	0.7279 (12)	0.5857 (15)	0.9907 (11)	0.027 (3)*	
H12	0.6818 (13)	0.4116 (16)	0.5460 (12)	0.034 (4)*	
H9	0.6088 (13)	0.5821 (17)	1.0245 (12)	0.036 (4)*	
H11	0.7788 (13)	0.4705 (16)	0.6362 (12)	0.032 (4)*	
H8	0.6298 (13)	0.6961 (18)	0.9372 (12)	0.035 (4)*	
H13	0.4820 (12)	0.3727 (17)	0.5783 (12)	0.034 (4)*	
H20	0.7076 (13)	0.3059 (16)	0.9306 (11)	0.029 (3)*	
H19	0.5963 (13)	0.3128 (18)	0.9806 (13)	0.041 (4)*	
H15	0.4790 (13)	0.5297 (17)	0.6277 (12)	0.034 (4)*	
H14	0.4571 (13)	0.3869 (16)	0.6953 (12)	0.034 (4)*	
H22	0.5884 (13)	0.2571 (18)	0.8637 (13)	0.038 (4)*	
H5	1.1462 (12)	0.6131 (15)	0.9024 (11)	0.028 (3)*	
H3	1.2709 (13)	0.6789 (17)	0.6323 (12)	0.037 (4)*	
H4	1.2746 (13)	0.7291 (18)	0.8131 (12)	0.040 (4)*	
H2	1.1388 (13)	0.5080 (17)	0.5413 (12)	0.034 (4)*	
H1	1.0101 (12)	0.3924 (16)	0.6328 (11)	0.027 (3)*	

### Atomic displacement parameters (Å^2^)

**Table:**

	*U*^11^	*U*^22^	*U*^33^	*U*^12^	*U*^13^	*U*^23^
P1	0.01307 (12)	0.01214 (12)	0.01264 (12)	−0.00065 (7)	0.00175 (8)	−0.00020 (7)
O2	0.0219 (3)	0.0150 (3)	0.0141 (3)	0.0025 (2)	0.0012 (2)	0.0012 (2)
N1	0.0134 (3)	0.0143 (3)	0.0154 (3)	0.0003 (3)	0.0031 (3)	0.0005 (3)
C1	0.0141 (4)	0.0135 (4)	0.0161 (4)	0.0019 (3)	0.0032 (3)	0.0015 (3)
C6	0.0229 (5)	0.0226 (5)	0.0214 (5)	−0.0063 (4)	0.0051 (4)	−0.0012 (4)
C12	0.0184 (4)	0.0157 (4)	0.0179 (4)	0.0004 (3)	0.0027 (3)	−0.0029 (3)
C13	0.0214 (5)	0.0297 (5)	0.0171 (4)	0.0020 (4)	0.0049 (4)	−0.0015 (4)
C2	0.0207 (4)	0.0222 (5)	0.0171 (4)	0.0005 (4)	0.0052 (3)	−0.0001 (3)
C4	0.0241 (5)	0.0279 (5)	0.0321 (6)	0.0004 (4)	0.0129 (4)	0.0102 (4)
C3	0.0275 (5)	0.0309 (6)	0.0209 (5)	0.0033 (4)	0.0110 (4)	0.0049 (4)
C5	0.0262 (5)	0.0280 (5)	0.0326 (6)	−0.0105 (4)	0.0077 (4)	0.0014 (4)
O1	0.0231 (3)	0.0169 (3)	0.0193 (3)	−0.0056 (3)	0.0003 (3)	−0.0021 (3)
C8	0.0148 (4)	0.0209 (4)	0.0182 (4)	0.0006 (3)	0.0064 (3)	0.0026 (3)
C9	0.0228 (5)	0.0247 (5)	0.0179 (4)	0.0034 (4)	0.0065 (4)	−0.0007 (4)
C11	0.0190 (4)	0.0267 (5)	0.0263 (5)	−0.0035 (4)	0.0005 (4)	−0.0067 (4)
C10	0.0306 (5)	0.0215 (5)	0.0264 (5)	−0.0017 (4)	0.0089 (4)	0.0066 (4)
O3	0.0137 (3)	0.0197 (3)	0.0161 (3)	0.0026 (2)	0.0023 (2)	−0.0017 (2)

### Geometric parameters (Å, º)

**Table:**

P1—O1	1.4932 (7)	C2—C3	1.3962 (14)
P1—O3	1.5191 (7)	C2—H1	0.948 (14)
P1—O2	1.5809 (7)	C4—C3	1.3845 (17)
P1—C1	1.8068 (9)	C4—C5	1.3910 (16)
O2—H6	0.847 (19)	C4—H3	0.949 (15)
N1—C8	1.5030 (12)	C3—H2	0.972 (15)
N1—C12	1.5077 (12)	C5—H4	0.957 (16)
N1—H17	0.900 (14)	C8—C10	1.5202 (14)
N1—H16	0.916 (15)	C8—C9	1.5208 (14)
C1—C2	1.3945 (13)	C8—H21	0.965 (13)
C1—C6	1.3966 (13)	C9—H7	0.989 (15)
C6—C5	1.3920 (14)	C9—H9	0.976 (15)
C6—H5	0.976 (14)	C9—H8	0.973 (16)
C12—C11	1.5188 (14)	C11—H13	0.987 (15)
C12—C13	1.5193 (14)	C11—H15	0.977 (16)
C12—H18	0.967 (13)	C11—H14	0.982 (15)
C13—H10	0.969 (15)	C10—H20	0.976 (15)
C13—H12	0.957 (15)	C10—H19	0.980 (16)
C13—H11	0.961 (15)	C10—H22	0.957 (16)
			
O1—P1—O3	115.20 (4)	C3—C4—C5	119.74 (9)
O1—P1—O2	109.72 (4)	C3—C4—H3	121.1 (9)
O3—P1—O2	109.58 (4)	C5—C4—H3	119.1 (9)
O1—P1—C1	108.51 (4)	C4—C3—C2	120.12 (10)
O3—P1—C1	108.10 (4)	C4—C3—H2	119.8 (9)
O2—P1—C1	105.24 (4)	C2—C3—H2	120.1 (9)
P1—O2—H6	114.8 (12)	C4—C5—C6	120.10 (10)
C8—N1—C12	117.16 (7)	C4—C5—H4	122.1 (10)
C8—N1—H17	106.5 (8)	C6—C5—H4	117.8 (10)
C12—N1—H17	110.1 (8)	N1—C8—C10	110.56 (8)
C8—N1—H16	107.3 (9)	N1—C8—C9	107.49 (8)
C12—N1—H16	107.5 (9)	C10—C8—C9	112.76 (9)
H17—N1—H16	107.9 (12)	N1—C8—H21	106.3 (7)
C2—C1—C6	118.58 (9)	C10—C8—H21	110.3 (8)
C2—C1—P1	121.24 (7)	C9—C8—H21	109.2 (7)
C6—C1—P1	120.17 (7)	C8—C9—H7	111.5 (8)
C5—C6—C1	120.74 (10)	C8—C9—H9	111.2 (9)
C5—C6—H5	120.4 (8)	H7—C9—H9	105.2 (12)
C1—C6—H5	118.8 (8)	C8—C9—H8	111.5 (9)
N1—C12—C11	110.17 (8)	H7—C9—H8	108.6 (12)
N1—C12—C13	107.55 (8)	H9—C9—H8	108.5 (12)
C11—C12—C13	111.46 (9)	C12—C11—H13	110.3 (8)
N1—C12—H18	107.9 (8)	C12—C11—H15	110.3 (9)
C11—C12—H18	110.2 (8)	H13—C11—H15	105.8 (12)
C13—C12—H18	109.5 (7)	C12—C11—H14	111.6 (9)
C12—C13—H10	110.1 (8)	H13—C11—H14	108.0 (12)
C12—C13—H12	109.8 (9)	H15—C11—H14	110.7 (12)
H10—C13—H12	107.5 (12)	C8—C10—H20	110.9 (8)
C12—C13—H11	111.6 (9)	C8—C10—H19	108.6 (9)
H10—C13—H11	108.2 (12)	H20—C10—H19	108.0 (12)
H12—C13—H11	109.6 (12)	C8—C10—H22	111.4 (9)
C1—C2—C3	120.72 (10)	H20—C10—H22	109.3 (12)
C1—C2—H1	119.7 (8)	H19—C10—H22	108.3 (13)
C3—C2—H1	119.6 (8)		
			
O1—P1—C1—C2	6.07 (9)	C8—N1—C12—C13	−179.89 (8)
O3—P1—C1—C2	−119.51 (8)	C6—C1—C2—C3	0.34 (14)
O2—P1—C1—C2	123.46 (8)	P1—C1—C2—C3	179.35 (8)
O1—P1—C1—C6	−174.93 (8)	C5—C4—C3—C2	−0.39 (17)
O3—P1—C1—C6	59.48 (8)	C1—C2—C3—C4	0.11 (16)
O2—P1—C1—C6	−57.55 (8)	C3—C4—C5—C6	0.21 (18)
C2—C1—C6—C5	−0.53 (15)	C1—C6—C5—C4	0.26 (17)
P1—C1—C6—C5	−179.55 (8)	C12—N1—C8—C10	58.07 (11)
C8—N1—C12—C11	58.44 (11)	C12—N1—C8—C9	−178.48 (8)

### Hydrogen-bond geometry (Å, º)

**Table:**

*D*—H···*A*	*D*—H	H···*A*	*D*···*A*	*D*—H···*A*
N1—H17···O3	0.900 (14)	1.960 (14)	2.8510 (10)	170.3 (12)
O2—H6···O3^i^	0.847 (19)	1.744 (19)	2.5895 (10)	177.4 (19)
N1—H16···O1^ii^	0.916 (15)	1.764 (15)	2.6782 (10)	176.7 (13)

Symmetry codes: (i) −*x*+2, −*y*+1, −*z*+2; (ii) −*x*+3/2, *y*+1/2, −*z*+3/2.
